# Long-term auxological and endocrinological evaluation of patients with 9p trisomy: a focus on the growth hormone-insulin-like growth factor-I axis

**DOI:** 10.1186/1472-6823-14-3

**Published:** 2014-01-08

**Authors:** Stefano Stagi, Elisabetta Lapi, Salvatore Seminara, Silvia Guarducci, Marilena Pantaleo, Sabrina Giglio, Francesco Chiarelli, Maurizio de Martino

**Affiliations:** 1 Department of Health’s Sciences, Paediatric Endocrinology Unit, University of Florence, Anna Meyer Children’s University Hospital, Florence, Italy; 2 Genetics and Molecular Medicine Unit, Anna Meyer Children’s University Hospital, Florence, Italy; 3 Department of Paediatrics, University of Chieti, Chieti, Italy

**Keywords:** Trisomy 9p, Growth Hormone, Growth hormone deficiency, Pubertal delay, Growth delay, Short Stature, Scoliosis

## Abstract

**Background:**

Trisomy 9p is an uncommon anomaly characterised by mental retardation, head and facial abnormalities, congenital heart defects, kidney abnormalities, and skeletal malformations. Affected children may also show growth and puberty retardation with delayed bone age. Auxological and endocrinological data are lacking for this syndrome.

**Methods:**

We describe three girls and one boy with 9p trisomy showing substantial growth failure, and we evaluate the main causes of their short stature.

**Results:**

The target height was normal in all families, ranging from 0.1 and -1.2 standard deviation scores (SDS). The patients had a low birth-weight (from -1.2 to -2.4 SDS), birth length (from -1.1 to -3.2 SDS), and head circumference (from -0.5 to -1.6 SDS). All patients presented with substantial growth (height) retardation at the time of 9p trisomy diagnosis (from -3.0 to -3.8 SDS).

The growth hormone stimulation test revealed a classic growth hormone (GH) deficiency (GHD) in patients 1, 3, and 4. In contrast, patient 2 was determined to have a GH neurosecretory dysfunction (GHNSD). The plasma concentrations of IGF-I and IGFBP-3 were low in all patients for their ages and sexes (from -2.0 to -3.4 SDS, and from -1.9 to -2.8 SDS, respectively).

The auxological follow-up showed that those patients who underwent rhGH treatment exhibited a very good response to the GH therapy, whereas patients 3 and 4, whose families chose not to use rhGH treatment, did not experience any significant catch-up growth.

**Conclusions:**

GH deficiency appears to be a possible feature of patients with 9p trisomy syndrome. These patients, particularly those with growth delays, should be evaluated for GH secretion.

## Background

Trisomy 9p is the fourth most frequent chromosome anomaly in live-born infants, after trisomies 21, 18, and 13 [1]. Since the original publication by Rethoré *et al.*[2], more than 150 patients with partial or complete 9p trisomy have been reported [1].

This disorder can occur because of a balanced chromosomal rearrangement between two chromosomes in one of the parents or as the result of a spontaneous (*de novo*) genetic change early in embryonic development occurring for unknown reasons [3-5].

In this disorder, part or all of the short arm (p) of chromosome 9 is duplicated [5]. In some cases, an extra (trisomic) segment of the long arm (q) of chromosome 9 may also be present [5].

The signs of this disorder are unusually similar among affected children, despite differing lengths of the duplicated segment. Patients with 9p trisomy exhibit mental retardation and characteristic head and facial abnormalities, such as microcephaly with large anterior fontanel and micrognathia, malformed protruding ears, hypertelorism, deep set eyes, and down slanting palpebral fissures [3,5]. Congenital heart defects [6,7], central nervous system abnormalities [8,9], kidney abnormalities [3,5], and skeletal malformations [10,11] are also present.

With respect to facial features, duplication of 9pter-p11 is associated with milder dysmorphia, whereas duplication of 9p21.1-q22-32 is associated with more severe craniofacial features [12].

Affected infants may also show growth retardation and delayed bone age [5]. However, very rarely has growth hormone (GH) deficiency (GHD) been reported [13].

Here, we studied the causes of short stature and investigated the GH insulin-like growth factor I (IGF-I) axis in patients with 9p trisomy during the follow-up of these patients, three of whom reached adult height.

## Methods

Four Caucasian subjects with 9p trisomy syndrome (1 boy and 3 girls, mean age at the diagnosis of 9p trisomy syndrome 19.5 months [range: 0.2 – 45.0]) who presented consecutively at the Genetics and Molecular Medicine Unit of Anna Meyer Children's University Hospital in Florence and were brought to us for delayed growth and development were evaluated.

A diagnosis of 9p trisomy syndrome was made for all subjects between 1994 and 2009 based on the clinical phenotype assessed by an experienced medical geneticist in our hospital and was confirmed by chromosome analysis.

All subjects underwent an extensive endocrine work-up to rule out possible causes of growth failure that included IGF-I, free-thyroxin (FT4), thyroid stimulating hormone (TSH), cortisol, glucose, electrolytes, venous blood gas, haemoglobin level, total protein, serum albumin, coagulation profile, calcium, phosphorous, alkaline phosphatase, vitamin D (25OHD), parathyroid hormone (PTH) and anti-tissue transglutaminase (tTG) screening tests for coeliac disease.

At the time of the auxological evaluation, all patients had a height less than 2 standard deviations (SD) below the mean for the normal population and/or a subnormal growth velocity.

In the absence of specific growth charts, age-related reference values for height, weight, and body mass index (BMI) were used [14]. As described, height, height velocity (HV), and BMI were normalised for chronological age by conversion to standard deviation scores (SDS). SDS were calculated according to the following formula: patient value - mean of the age-related reference value/standard deviation of the age-related reference value [15].

In all subjects, we studied the GH response to GH stimulation tests (oral clonidine and/or arginine and/or GH releasing hormone (GHRH) + arginine test).

A GHD diagnosis was made based on auxological criteria (height and/or growth velocity 2 SD below the mean for the normal population) and a GH peak below 10 μg/L (below 20 μg/L after the GHRH + arginine test) after two tests were conducted on two separate occasions [16].

In the event of growth failure with low serum IGF-I and normal GH concentrations, after pharmacological treatment, assessment of spontaneous 12-hour (12 hr) GH secretion was performed [17]. In this case, GH profiling at 30-min intervals over 12 hr was performed. GH neurosecretory dysfunction (GHNSD) was diagnosed when the 12-hr integrated concentrations of GH were 3.2 μg/litre or less [17].

Pubertal stage was assessed according to Marshal and Tanner [18], and testicular volume was determined with the Prader orchidometer. The age of pubertal onset was defined as the age at durable Tanner B2 stage for females and a testicular volume of more than 4 ml for males (G2). The age at which the onset of puberty occurred was recorded as the average age between the previous clinic visit, when the child was still prepubertal, and the clinic visit when the child was G2/B2. The duration of puberty was recorded as the time from G2/B2 to G4/B4. The age at G4/B4 was assessed by averaging the ages at the previous clinic visit, when the child was G3/B3, and the clinic visit when the child was G4/B4. Pubertal growth was defined as growth occurring between the onset of puberty and the FH.

When the onset of puberty occurred, we studied the luteinizing hormone (LH) and follicle stimulating hormone (FSH) response to gonadotropin releasing hormone (GnRH) test, estradiol or total testosterone levels, and we performed in the females a pelvic ultrasound; at onset of puberty a normal GnRH test was defined as a peak LH value > 6 IU/L. However, during pubertal follow-up, estradiol or testosterone levels and/or pelvic ultrasound were also carried out.

Bone age (BA) was evaluated through radiographs of the left hand and wrist and then calculated according to the Greulich and Pyle method [19]. Predicted adult height (PAH) was determined using BA, calculated according the Greulich and Pyle method, and height, calculated according to the Bayley-Pinneau method [20]. Target height (TH) was estimated according the method of Hermanussen and Cole [21] by calculating the midparent height as an SDS and correcting this by a factor corresponding to the influence of assortative mating and parent-offspring correlations.

In these patients, anthropometric data were collected periodically (near every 6 months to 1 year). Near- and/or final height (FH) was commonly defined, recognising that children may continue to grow after achieving this definition, for patients in whom both the chronological age (CA) and BA were at least 14 yr for females and 16 yr for males [15]. Because 9p trisomy patients have been reported to continue to grow for a long time, the near-adult height (NAH) was defined for all female and male patients with a CA of at least 18 and 20 yr, respectively, with an HV < 1 cm/year for two consecutive years.

Intracranial imaging was obtained by magnetic resonance imaging (MRI) using precontrast coronal spin echo T1-weighted images, followed by post-gadolinium T1-weighted imaging.

The study was approved by the Anna Meyer Children’s University Hospital ethics committee, and informed consent was obtained from the children’s parents.

### Laboratory methods

Glucose, electrolytes, creatinine, venous blood gas, haemoglobin level, total protein, serum albumin, coagulation profile, calcium, phosphorous, alkaline phosphatase, 25OHD, and PTH were measured using standard tests.

Free-T4 and uTSH serum levels were determined by immunometric assays (ImmuliteTM 2000 Third Generation, DPC Diagnostic Products Corporation, Los Angeles, CA, USA). The within- and between-run coefficients of variation were less than 12.5% for uTSH and less than 7.5% for FT4.

The IgA-tTG antibody testing was performed using commercially available ELISA kits (The Binding Site Limited, Birmingham, UK). The ELISA was performed in duplicate for all of the sera samples, as per the manufacturer’s instructions.

The serum total IGF-I levels were measured using a solid-phase enzyme-labelled chemiluminescent immunometric assay on the Immulite 2000 automated immunoanalyser (Siemens Medical Solutions Diagnostics, Los Angeles, CA, USA) with an inter-assay coefficient of variation of ~4%. Serum GH was analysed by IMMULITE 2000 Growth Hormone, an automated chemiluminescence enzyme immunoassay analyser. The intra-assay and inter-assay variability coefficients for this kit at the decision limits were 2.5% and 3.8%, respectively.

LH, FSH, cortisol, estradiol and total testosterone were measured by a chemiluminescent immunometric assay (Immulite 2000, Third Generation, DPC Diagnostic Products Corporation, Los Angeles, CA, USA). The intra-assay and inter-assay variability coefficients for this kit at the decision limits were 3.6%, 2.1%, 4.8%, 6.4% and 5.8% respectively.

#### Chromosome analysis

For the diagnosis of 9p trisomy, chromosome analysis using GTG-banding was performed according to standard procedures [22]. A total of 20 metaphases from stimulated peripheral blood cultures were analysed. Karyotypes were described according to the International System for Human Cytogenetic Nomenclature [23].

Fluorescence in situ hybridisation (FISH) was performed according to the manufacturer’s instructions using a locus specific probe LSI 9p21, a subtelomeric (ST) probe for the short arm of chromosome 9 and a chromosome enumeration probe (CEP) 9 (Abbott Molecular/Vysis, USA).

When possible, CGH array analysis was performed using the Agilent Human Genome CGH Microarray Kit 44 K (Agilent Technologies, Santa Clara, California, USA). This platform is a high-resolution oligonucleotide-based microarray with a resolution of approximately 150 kb. Labelling and hybridisation were performed following the protocols provided by Agilent: 500 ng of purified DNA from the patient and 500 ng of control purified DNA of the same sex (Agilent) were double digested with RsaI and AluI enzymes (Agilent) for 2 h at 37°C, which resulted in products between 200 bp and 500 bp in length. Each digested sample was labelled for 2 h, minimising light exposure, using the Agilent Genomic DNA Labeling Kit, using Cy5-dUTP for the patient DNA and Cy3-dUTP for the reference DNA. Labelled products were column purified (Agilent) and prepared by combining the test and control samples according to the Agilent protocol. After probe denaturation and pre-annealing with 50 μg of human Cot-1 DNA (Invitrogen), hybridisation was performed at 65°C for 24 h in a rotating oven at 20 rpm. After two washing steps, the array slide was scanned with the Agilent C Scanner. The spot intensities were measured, and the image files were quantified using the Agilent Feature Extraction 10.5 software. Text outputs from the quantitative analyses were imported into Genomic Workbench Standard Edition 5.0 software (Agilent Technologies). Breakpoint positions were reported according to Hg 18, build 36 [24,25].

## Results

The main characteristics of our study are reported in Tables 1 and 2. Three patients were followed-up from the age of 9p trisomy diagnosis until their final or near final height (FH). One patient was followed-up for two years after the GHD diagnosis.

**Table 1:** General, auxological and endocrinological characteristics of our patients

**Main characteristics**		**Case 1**	**Case 2**	**Case 3**	**Case 4**
Sex (Female: Male)		F	F	M	F
Parental age, yrs^ (Mother: Father)		25:26	33:36	25:28	34:36
Target height, cm (SDS)		162.6 (0.1 SDS)	159 (-0.5 SDS).	166.8 (- 1.2 SDS)	159.5 (-0.5 SDS)
Gestation (weeks)		uncomplicated (41)	uncomplicated (40)	uncomplicated (40)	uncomplicated (39.4)
Birth-weight, gr (SDS)		2.650 (-2.4)	2.850 (-1.6)	3.140 (0.3 SDS)	2.370 (-1.6 SDS)
Birth-length, cm (SDS)		45 (-3.2)	46 (-2.4)	49 (-0.4 SDS)	45 (-2.4 SDS)
OFC, cm (SDS)		34 (-0.5)	-	33 (-1.1 SDS).	32 (-1.6 SDS)
Neuromotor development		Delayed	Moderately delayed	Delayed	Delayed
9p trisomy diagnosis		At birth	7 months	3 years 9 months	2 years 2 months
Chromosomal study		47,XX, t (9; 14) (q12; p11.1), +der (9)t(9;14) (q12; p11.1) [24]	46,XX,-9, dup (9) (p12; p22) [24]	46,XY, der (22)t(9;22)(p21; p12). [24]	46,XX, dup(9)(p24.3-p13.3)
Postnatal growth delay		Yes	Yes	Yes	Yes
GH stimulation tests (type)*		(C) - (A)	(A) - (C)	(GA) - (C)	(C) - (GA)
	Basal GH level (ng/mL)	0.9 - 1.3	1.7 - 0.2	0.7 - 1.1	0.4 - 0.5
	Peak GH level (ng/mL)	7.0 - 7.4	5.0 – 13.0	6.3 – 4.3	9.6 – 8.8
GH axis dysfunction type		GHD	GHNSD	GHD	GHD
GH treatment (Y:N)		Yes	Yes	No	No
Puberty onset, B2 or G2 (yrs)		11.2	14.5	14.8	-
	B3 or G3	11.9	15.6	16.2	-
	B4 or G4	12.9	16.9	17.5	-
Final height, cm (SDS)		156.9 (-0.9 SDS).	154.4 (-1.3 SDS)	157.5 (-2.6 SDS)	-

OFC: Occipito-Frontal head Circumference; GH: Growth Hormone; GHD: Growth Hormone Deficiency; GHNSD: Growth Hormone Neurosecretory Dysfunction.

* (A) = arginine; (C) = clonidine; (GA) = GHRH + arginine; ^At conception.

**Table 2:** Comparison of our patients’ phenotypes with those reported in the literature

**Phenotype**	**Literature (%)**	**Case 1**	**Case 2**	**Case 3**	**Case 4**
Downturned oral commissures	95%	+	+	+	+
Bulbous nose	95%	+	+	+	+
Malformed/low-set ears	70-80%	+	+	+	+
Strabismus	70-80%			+	-
Short philtrum	70-80%	+	+	+	+
Hypertelorism	70-80%	+	+	+	-
Microcephaly	70-75%	-	-	+	-
Brachycephaly	70-75%	+	+	+	-
Cleft lip/palate	5%	-	-	-	-
Enophthalmos	60-70%	+	-	-	-
Ogival palate	60-70%	ND	+	+	-
Down-slanting palpebral fissures	60-70%	+	+	+	+
Growth retardation	99%	+	+	+	+
Delayed skeletal maturation	99%	+	+	+	+
Delayed puberty	70-90%	-	+	+	ND
Low birthweight	50-70%	+	+	-	-
Clinodactyly	90%	+	+	+	+
Brachydactyly	90%	+	+	+	-
Nail hypoplasia	70-75%	+	-	+	+
Lordosis	60%	+	+	-	-
Kyphoscoliosis	60%	+	+	+	-
Short neck	60-70%	+	-	+	-
Single palmar crease	80-95%	+	+	+	-
Single crease of the fifth finger	30-50%	+	+	+	-
Mental retardation	60%	+	+	+	+
Language delay	90%	+	+	+	+
Hypotonia	60-70%	+	+	+	-
Agenesis/hypoplasia of the corpus callosum	< 30%	agenesis	hypogenesis	agenesis	ND
Epilepsy	< 30%	-	+	-	-
Ventriculomegaly	< 30%	+	-	-	-
Cerebral hypoplasia	< 30%	-	-	-	-
Cardiac anomalies	20%	-		-	-
Umbilical hernia	rare	-		-	-
Other signs	-		spondylolisthesis	urogenital anomalies	

ND: not determined.

The family history was unremarkable for all of the subjects in this study. Three patients were only children of healthy non-consanguineous Italian parents. One female, the second child, had an older sister with coeliac disease. None of the parents had short stature or a positive history for pubertal delay, except for the mother of patient 1, whose menarche was at the age of 14. The target height was normal in all families, ranging from 0.1 SDS and -1.2 SDS.

There was no apparent parental age effect (Table 1). All patients were born at term following uncomplicated gestations. The patients had a low birth-weight (from -1.2 to -2.4 SDS), birth length (from -1.1 to -3.2 SDS), and head circumference (from -0.5 to -1.6 SDS).

**Figure 1:**
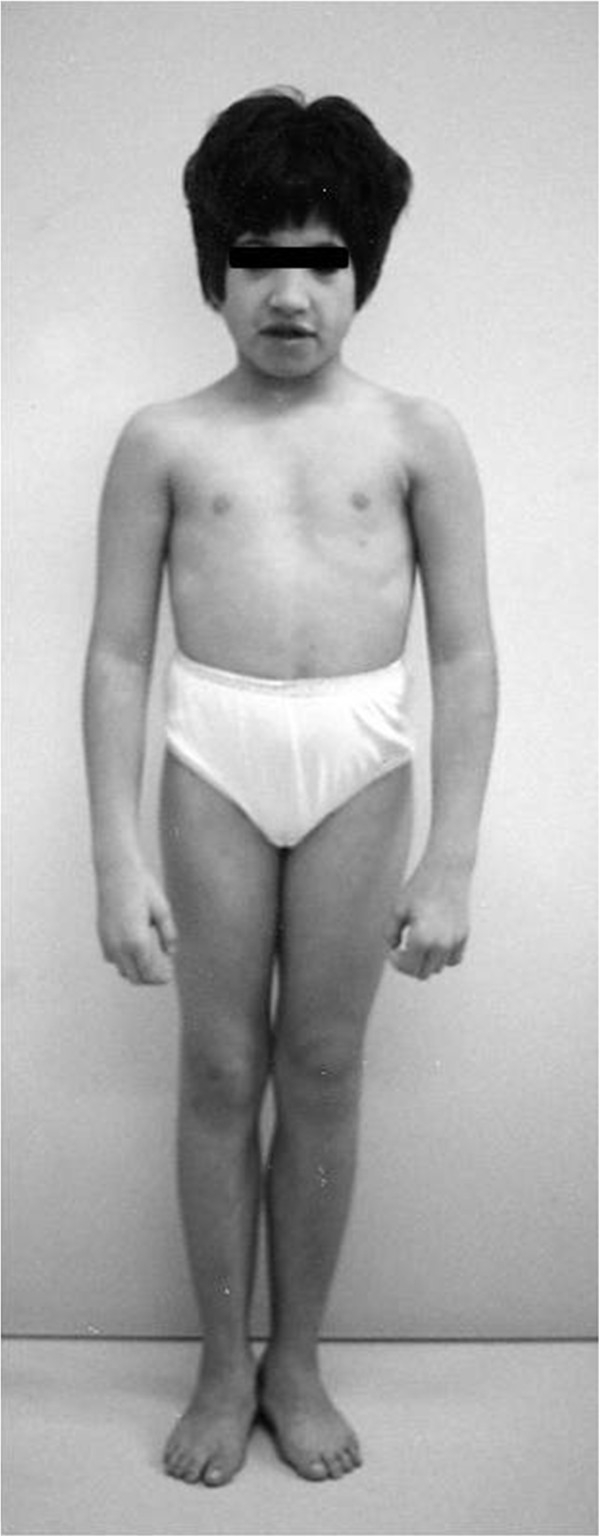
**Some typical facial dysmorphisms of trisomy 9p syndrome.** AP of case 1 at 7 years of age. The patient showed facial dysmorphisms typical of trisomy 9p syndrome, such as brachycephaly, hypertelorism, down-slanting palpebral fissures, broad prominent nose, and large low-set ears.

The facial gestalt in our 9p trisomy syndrome was striking. Typically, all patients showed significant craniofacial characteristics, such as brachycephaly, hypertelorism, down-slanting palpebral fissures, a prominent nose with a globous tip, a wide philtrum, large low-set ears, and a short and broad neck (Figure 1). In addition, two patients experienced frequent infections of the upper airways, bronchitis, and bronchiolitis. Three patients exhibited kyphoscoliosis; of these patients, one also exhibited spondylolisthesis.

At the first evaluation, auxological data showed that all patients presented with substantial growth retardation at the time of 9p trisomy diagnosis for height (from -3.0 to -3.8 SDS), whereas the BMI was in the normal range (from 0.0 to -0.9 SDS). At the time of diagnosis, bone age, determined by Greulich and Pyle’s method, was always considerably delayed (from – 1 years at 2 years and 4 months, to -2 yrs 8 months at 5 years 6 months).

Endocrine evaluation revealed normal thyroid function, TSH and cortisol level. However, tTG was negative in all patients. Growth hormone stimulation tests revealed a classic GHD in patients 1, 3, and 4. In contrast, patient 2 was determined to have a GHNSD (Table 1). The plasma concentrations of IGF-I and IGFBP-3 were low in all patients for their ages and sexes (from -2.0 to -3.4 SDS, and from -1.9 to -2.8 SDS, respectively). Patients 3 and 4 were not treated with rhGH as a result of their family’s decisions. None of the patients showed multiple pituitary hormone insufficiencies.

The MRI disclosed agenesis of the corpus callosum in patients 1 and 3, whereas patient 2 showed hypoplasia of the corpus callosum (Table 2). All patients showed normal hypophysis with respect to size and structure.

**Figure 2:**
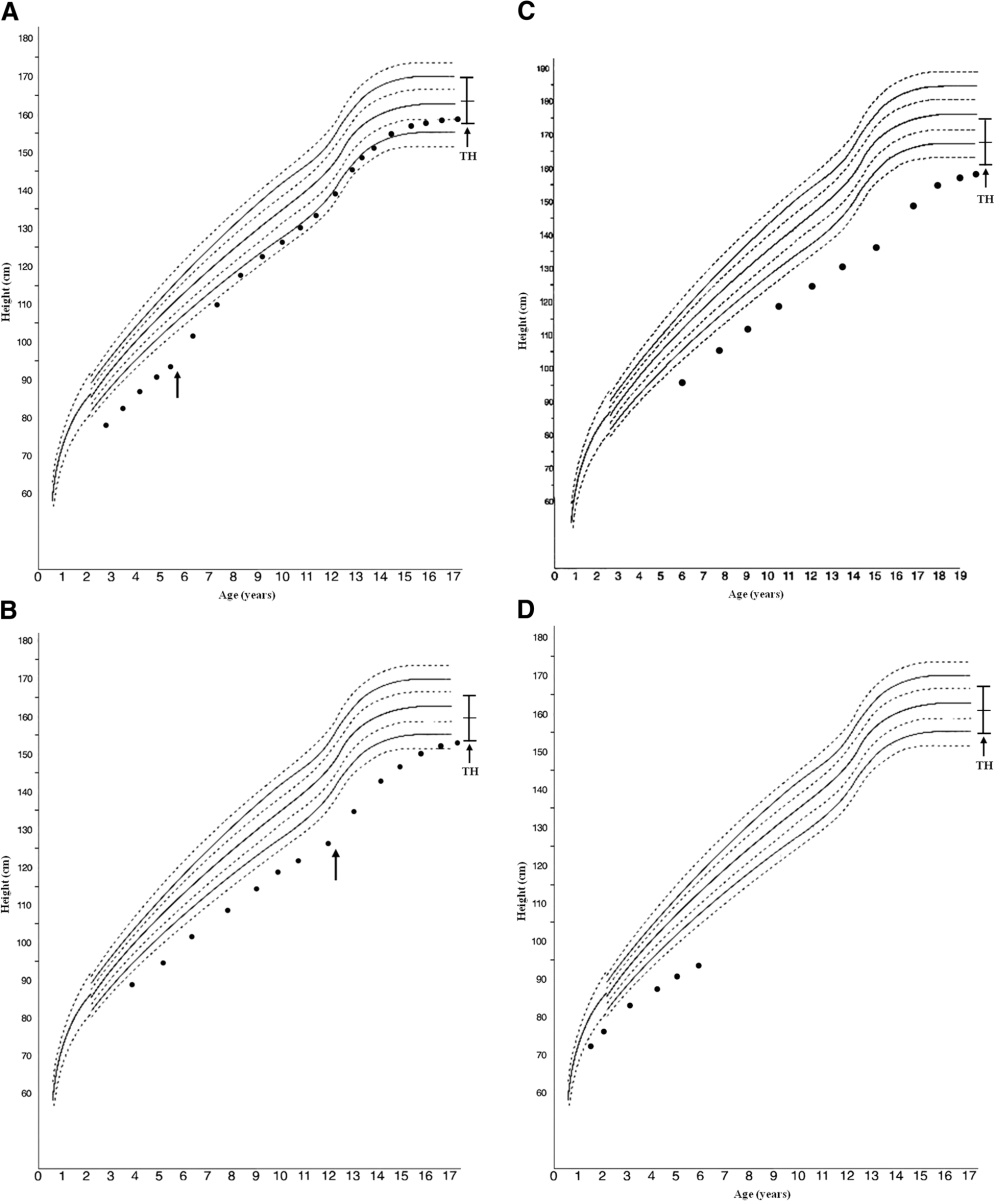
**Growth charts of the four patients. A)** case 1, **B)** case 2, **C)** case 3, and **D)** case 4. The arrow indicates the age of onset of growth hormone treatment.

The auxological follow-up showed that all rhGH-treated patients (0.22 mg/kg per week subcutaneously) had a very good response to the GH therapy (Figure 2a and b), with an SDS-GV that increased remarkably during therapy, whereas patients 3 and 4 did not experience significant catch-up growth (Figure 2c and d). As expected, in rhGH-treated patients IGF-I (SDS) increased significantly after 12 and 24 months of treatment (1.1 ± 0.8 and 1.3 ± 1.1, respectively; P < 0.001).

During the rhGH treatment, one patient exhibited a worsening of scoliosis, which however required only a medical treatment, whereas the degree of scoliosis was not changed in another rhGH treated patient or in a rhGH untreated patient.

Pubertal onset was always delayed, except in the patient 1 (Table 1), possibly due to a different age of the start of rhGH treatment. The hormonal pattern of the hypophysis-ovary axis was normal in the three patients who were evaluated. Endocrine evaluation at the age of onset of puberty revealed a pubertal response of gonadotropins in all patients, with normal estradiol or testosterone levels. The follow-up analysis of pubertal development was also normal in these patients. During follow-up, estradiol or testosterone levels were in accordance to the pubertal stages.

The final height was consistent with the target height for those patients who started rhGH treatment; however, patient 3 had a final height that was considerably lower than the target height. The extents of the height response and recovery appeared to be related to the earlier age of start of the rhGH treatment.

**Figure 3:**
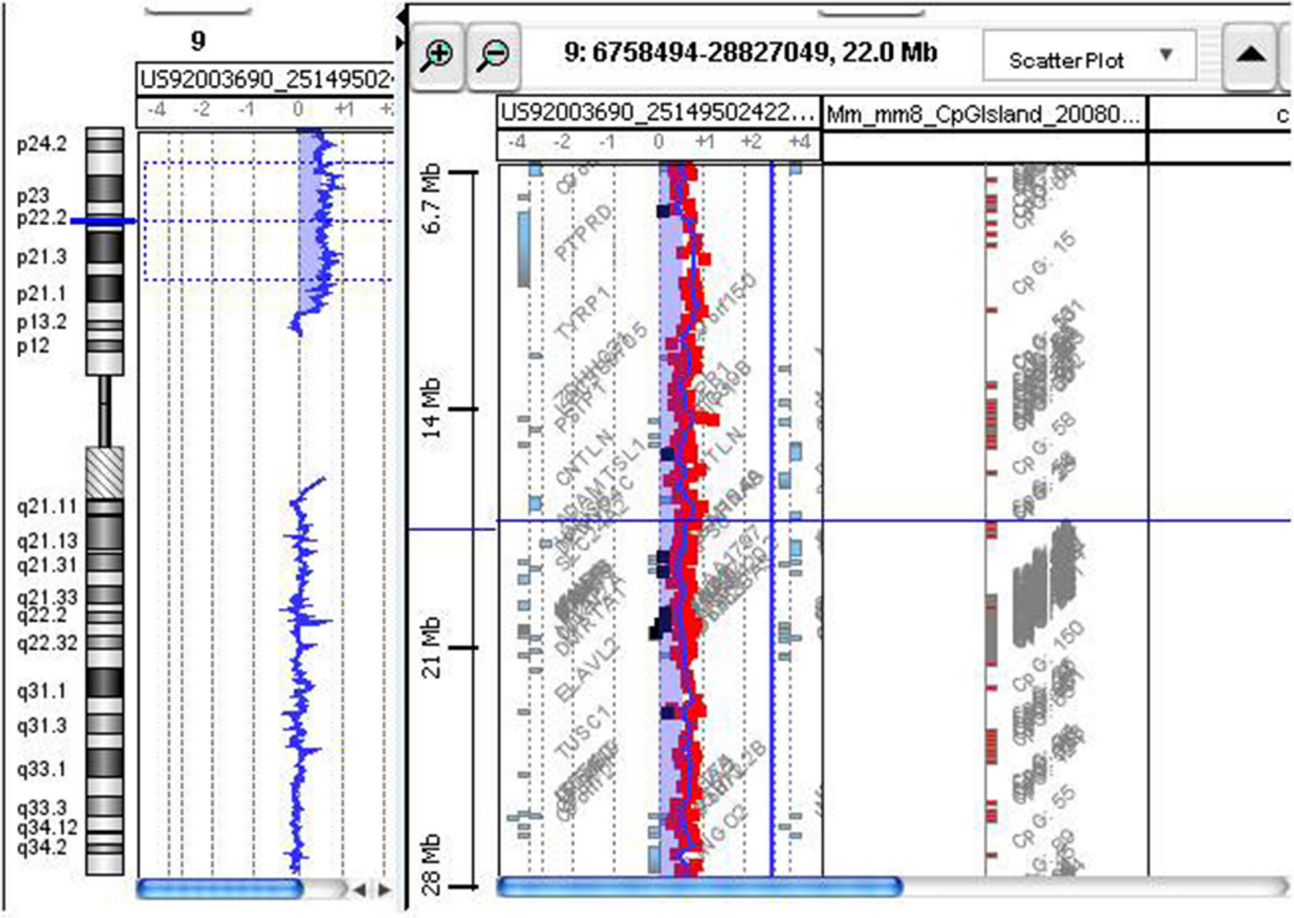
**Molecular karyotyping was performed by array-CGH on the proband’s DNA using an Agilent 44 K array platform with a resolution of approximately 100 kb.** Based on the physical mapping positions of the March 2006 Assembly (NCBI36/hg18) of the UCSC Genome Browser, this analysis showed a duplication of approximately 34 Mb that involved the 9p24.3-p13.3 region, with the breakpoint falling between 601,628 bp (first duplicated oligomer) and 34,638,095 bp (last duplicated oligomer).

Finally, in all patients, diagnoses were made based on chromosomal studies. Array-CGH analysis was conducted only for patient 4 and demonstrated a large duplication of near 34 Mb del 9p24.3-p13.3 (Figure 3). The other three families refused the array-CGH analysis [26].

## Discussion

Since the first description of trisomy 9p in 1970, there has been a rapidly increasing recognition and reporting of new cases [3,5]. In these patients, the spectrum of physical features is remarkably consistent, despite the varying sizes of the duplicated segments [27]. However, growth and endocrine function in patients with 9p trisomy syndrome have been scarcely evaluated [5,13].

Our data confirm that delays in growth and development can be important features in many patients with 9p trisomy [1; 27]. Nevertheless, our study showed that growth abnormalities might imply a growth deficiency of both postnatal [27], and prenatal onset.

Our data further confirm that bone maturation was significantly delayed in all patients [27] and that many of them may continue to grow up to the age of 20 and thus may partially catch up, even if their adult height may be reduced in comparison with their target height, as was the case in patient 3, who was not treated with hrGH [28].

Interestingly, our data show that this syndrome may be associated with neurological abnormalities, such as hypogenesis or agenesis of the corpus callosum [1]. In fact, in two of our patients, we observed agenesis and hypogenesis of the corpus callosum, congenital midline cerebral abnormalities that are frequently observed together with a GH deficiency or other disorders of the hypothalamic-pituitary axis [29-31]. Observations of brain malformations in these patients indicate that a brain MRI should be routinely considered in subjects with this disorder [1].

Our study strongly suggests that an impairment of the GH – IGF-I axis appears to be another possible feature of the trisomy 9p syndrome, and these patients should be tested for GH secretion, particularly patients with trisomy 9p syndrome with growth delay and midline cerebral malformations.

The response to GH treatment was good in patients 1 and 2; patient 2 exhibited a normal response to the GH stimulus tests and a neurosecretory pattern at the borderline of normality. So, GH treatment could also be considered to ameliorate the stature prognosis in patients with trisomy 9p and borderline values for the GH dynamic tests. Nevertheless, further studies will have to confirm the characteristics of the GH-IGF-I axis in patients with trisomy 9p syndrome.

Our study also confirms that patients with trisomy 9p syndrome show a delayed onset of puberty. In these patients, gonadotropin, thyroid and adrenal function appear to be normal. The cause of this disorder is not clear: it could be a peculiarity of the trisomy 9p syndrome, but it may also be attributable to the important delay in bone age, as evidenced in patients 2 and 3. Patient 1 had normal pubertal development, which is likely due to the precocious GH treatment, which normalised the bone age/chronological age ratio. In fact, a delayed onset of puberty, in the absence of gonadotropin deficiency, is a possible symptom in late-diagnosed cases of GH deficiency [32]. Therefore, an undiagnosed GH deficiency may partially explain the pubertal delay in these patients.

Patients with trisomy 9p syndrome also show common skeletal abnormalities, particularly scoliosis [10,11], which usually develops during the second decade [27]. GH treatment has been described as a factor that increases the risk of the progression of scoliosis [33], even if the results of other studies do not permit one to conclude that a relation exists between GH treatment and scoliotic progression [34]. Therefore, children at risk for scoliosis require close monitoring during GH therapy [35], as our patients 2 and 3 (who had scoliosis and vertebral schisis). A worsening of scoliosis in response to GH treatment was especially apparent in patient 2; however, this patient was not required the stop GH treatment. Thus, patients with 9p trisomy and GH deficiency should also be carefully evaluated for this problem.

## Conclusion

In conclusion, our study shows that GH deficiency or GHND may be diagnosed frequently, and an impairment of the GH-IGF-I axis may be another feature of patients affected by trisomy 9p syndrome, suggesting a possible cause of their growth and puberty delays. In our rhGH-treated patients, this therapy turned out to be effective. However, further studies are necessary to evaluate growth and puberty patterns in this syndrome.

The parents of the patients provided written informed consent for publication of individual clinical details.

## Competing interests

The authors declare that they have no competing interests.

Financial competing interests: The Authors have any financial and non-financial competing interests in relation to this manuscript.

## Authors’ contributions

SS carried out the endocrinological evaluation, conceived of the study and participated in its design. EL, carried out the clinical genetic diagnosis. SS carried out the endocrinological evaluation. SG carried out the molecular genetic studies. MP carried out the molecular genetic studies. SG carried out the clinical genetic diagnosis. FC participated in the endocrinological evaluation. MM participated in the endocrinological evaluation and participated in its coordination. All authors read and approved the final manuscript.

## Pre-publication history

The pre-publication history for this paper can be accessed here:

http://www.biomedcentral.com/1472-6823/14/3/prepub
